# Oxidative stress and inflammatory process in borderline personality disorder (BPD): a narrative review

**DOI:** 10.1590/1414-431X2023e12484

**Published:** 2023-03-17

**Authors:** A.R.C.C. Forte, P.H.C. Lessa, A.J.M. Chaves, P.E.A. de Aquino, L.M. Brito, L.C. Pinheiro, M.F. Juruena, D.F. de Lucena, P.H.F. de Rezende, S.M.M. de Vasconcelos

**Affiliations:** 1 Laboratório de Neuropsicofarmacologia, Universidade Federal do Ceará, Fortaleza, CE, Brasil; 2 Curso de Medicina, Departamento de Ciências Biológicas e da Saúde (DCBS), Universidade Federal do Amapá, Macapá, AP, Brasil; 3 Institute of Psychiatry, Psychology and Neuroscience, King's College London, London, UK

**Keywords:** Borderline personality disorder (BPD), Neuro-inflammation, BDNF, cytokines, Oxidative stress

## Abstract

Borderline personality disorder (BPD) is a severe psychiatric condition that affects up to 2.7% of the population and is highly linked to functional impairment and suicide. Despite its severity, there is a lack of knowledge about its pathophysiology. Studies show genetic influence and childhood violence as factors that may contribute to the development of BPD; however, the involvement of neuroinflammation in BPD remains poorly investigated. This article aimed to explore the pathophysiology of BPD according to the levels of brain-derived neurotrophic factor (BDNF), inflammatory cytokines, and oxidative stress substances that exacerbate neuronal damage. Few articles have been published on this theme. They show that patients with BPD have a lower level of BDNF and a higher level of tumor necrosis factor (TNF)-α and interleukin (IL)-6 in peripheral blood, associated with increased plasma levels of oxidative stress markers, such as malondialdehyde and 8-hydroxy-2-deoxyguanosine. Therefore, more research on the topic is needed, mainly with a pre-clinical and clinical focus.

## Introduction

Borderline personality disorder (BPD) is a severe psychiatric disorder that results in high suicide rates, functional and occupational impairment, and intensive medical and psychiatric treatment, creating high social costs (1). BPD involves rapid and intense emotional fluctuations, inconsistent and unstable identity, and troubled interpersonal relationships (2). Two recent studies suggest a prevalence of BPD of 0.7 to 1.2% and 0.7 to 2.7% in adults (3,4). However, in individuals with psychiatric disorders (outpatient and in-hospital) BPD varies from 15 to 28% (5). In psychiatric emergency units, patients with BPD account for 10 to 15% of medical care (6) and represent 6% of primary care medical appointments (7). Often, patients do not receive a proper BPD diagnosis because they are not evaluated by a mental health professional. Consequently, they are not referred to the most appropriate treatments.

Although BPD is a public health problem, there is a lack of knowledge about its pathophysiology. Some studies suggest that mental disorders have an important relationship to neurotrophin dysregulation (8), inflammatory markers (9), and oxidative stress (10). There are papers that show higher levels of some cytokines and oxidative stress markers in psychotic patients (11), anxious patients (12), bipolar patients (13), and attention deficit hyperactivity disorder (ADHA) patients (14).

A combination of factors influences the development of BPD (15), including genetic heritability (16) and childhood violence (17). Other variables include emotional abuse and negligence (18), in addition to low parental care or excessive mother care (19). These adversities influence children's development (20) and trigger stress response (21). As stress is a risk and an aggravating factor for BPD, being associated with systemic inflammation (13), inflammation may also be a cause and a consequence of BPD.

Furthermore, it has been shown in animal models that, in some brain regions, such as hippocampus, prefrontal cortex, and amygdala, alterations in levels of brain markers are related to development of mental disorders (22), such as depression (23) and schizophrenia (24).

Considering that BPD is a disorder that causes intense individual and family suffering, this paper intends to make a narrative review about BPD and its relationship to neurotrophins, inflammatory markers, and oxidative stress.

## Material and Methods

A literature search was conducted in Pubmed, Scielo, and Cochrane databases. The timeframe for publications was from 1960 to 2022 due to the lack of papers in the area. The term “borderline personality disorder” was mandatory, and the terms “oxidative stress”, “neurotrophin”, “inflammation”, “inflammatory cytokines”, or “BDNF” were flexible. Papers in English language regarding clinical and experimental approaches involving BPD, peripheral inflammatory markers, oxidative stress, and brain-derived neurotrophic factor (BDNF) composed the inclusion criteria (Table 1). Studies on bipolar disorder as well as approaches that did not provide data of inflammatory biomarkers, oxidative stress markers, and BDNF were excluded.

**Table 1:** List of studies that link borderline personality disorder (BPD) with neurotrophins, inflammatory cytokines, or oxidative stress.

Reference	Goals	Methodology	Results
Koenigsberg et al. 2012 (40)	Analyze BDNF levels in patients with BPD.	Analysis of BDNF platelet levels in 24 patients with BPD (33% females/age: 37.9 (9.2)), compared to 18 healthy controls (38% females, age: 29.9 (7.4)).	Decreased BDNF level in platelets of male patients with BPD (P<0.01).
Perroud et al. 2013 (41)	Investigate methylation status of exons I and IV in BPD patients and BDNF levels.	Blood analysis of 115 patients with BPD (93.91% females/age: 30.36 (9.19)) and compared to 52 controls (46.15% females/age: 40.65 (12.04)).	Higher BDNF level in plasma of patients with BPD (P<0.01).
Kahl et al.2005 (49)	Investigate bone density and immune markers in women with BPD.	Measurement of immune markers in blood of 16 women with BPD (age: 25.9 (5.0)), 12 women with BPD and depression during their lifetime (age: 31.8 (6.5)), and 10 women with BPD and depression currently facing the pathology (age: 24.2 (5.9)) compared to 20 healthy women (age: 26.1 (5.1)).	Higher levels of TNF-α and IL-6 in patients with BPD and current depression (P<0.05).
Kahl et al. 2006 (50)	Examine plasma cortisol profile, cytokines and cortisol/DHEA ratio in patients with BPD and depression.	Serum level analysis of cortisol, DHEA, TNF-α, IL-6, IL1β in 12 women with BPD and depression (age: 25.6 (3.9)), compared to 12 healthy women (age: 26.3 (5.1)).	Higher levels of TNF-α and IL-6 in patients with BPD and depression (P<0.05).
Lee et al. 2020 (66)	Investigate if individuals with personality disorders had changes in 8-OH-DG concentration.	Measurement of 8-OH-DG blood levels in 98 individuals with mental disorders: 43 with BPD (68.3% females/age: 36.33 (8.64), 29 with mental disorders other than personality disorders (65.5% females/age: 35.76 (6.26)) compared to 68 healthy controls (55% females/age: 32 (9.08)).	Higher levels of 8-OH-DG in whole blood of patients with BPD (P<0.01).
MacDowell et al. 2020 (67)	Investigate changes in inflammatory and oxidative responses in patients with BPD.	Measurement of serum levels of TBARS, nitrites, and antioxidant enzymes and investigation of intracellular components involved withinflammation and oxidation (IκBα, NFκB, iNOS, COX2, Keap1, NQO1, and HO1) in 49 patients with BPD (87.8% females/age: 29.8) and 33 healthy controls (93.8% females/age: 30.5)).	Decreased levels of antioxidant enzymes (CAT, GPx, and SOD) and IκBα in serum in patients with BPD. Increase of inflammatory factors (NFκB, iNOS, COX2, Keap1, NQO1) in peripheral blood mononuclear cells in those patients (P<0.05).

Age is reported as mean (standard deviation) in years. BDNF: brain-derived neurotrophic factor; BPD: borderline personality disorder; DHEA: dehydroepiandristerone; TNF-α: tumor necrosis factor alpha; IL-6: interleukin 6; IL1β: interleukin 1-beta; 8-OH-DG: 8-hydroxyguanosine; TBARS: thiobarbituric acid reactive substances; IκBα: nuclear factor of kappa light polypeptide gene enhancer in B-cells inhibitor alpha; NFκB: nuclear factor kappa light chain enhancer of activated B cells; iNOS: nitric oxide synthase; COX2: cyclooxygenase-2; Keap1: Kelch-like ECH-associated protein 1; NQO1: human NADPH quinone oxidoreductase; HO1: heme oxygenase-1; CAT: catalase; GPx: glutathione peroxidase; SOD: superoxide dismutase.

## Neuroimmune mechanisms involved in BPD

### Neurotrophins

Neurotrophins are molecules that provide neuron survival and development, define cell and systemic functions, and promote neuroplasticity (25,26). After the discovery of the first substance in 1950, the neuronal growth factor (NGF) (27), other neurotrophin have been identified, such as BDNF, neurotrophin-3 (NTF-3), and neurotrophin-4 (NTF-4) (28). Each of these molecules links its structures to tropomyosin-related receptors TrkA, TrkB, or TrkC (29).

Among them, BDNF is the best-studied molecule (30). Its fundamental role in learning and memory processes (31) and in cognitive and behavioral functions related to personal mood are well known (8).

BDNF synthesis occurs with the help of a precursor protein called proBDNF, which is stored in axons and dendrites (32) and induces its proteolytic cleavage in intra- or extracellular spaces (33,34). This produces a mature molecule that may cause physiological changes. BDNF binds to the TrkB receptor when in contact with neural tissues, causing dimerization due to neuron depolarization, with calcium influx through voltage-dependent calcium channels or NMDA-type calcium-permeable glutamate receptors, triggering intracellular signaling that leads to DNA transcription and cell response.

In this way, damage to structural or functional neural areas - such as amygdala (responsible for feelings such as fear and arousal), hippocampus (responsible for reward and learning comprehension), and orbitofrontal cortex (responsible for decision making and reward processing) - predisposes patients to the development of psychiatric disorders (35). Indeed, BDNF concentration changes in neural areas became an essential research element on brain disorders due to high BDNF concentration and expression in the amygdala, hippocampus, and brain cortex (22).

Central nervous system (CNS) BDNF levels can be predicted by BDNF level in peripheral blood (36). This was an important discovery because of the difficulty to measure BDNF in live human brains and because of the BDNF capacity to cross the blood-brain barrier in its free form (not bound to other substances or receptors) in either direction (37). As the production of BDNF in cells other than neurons and glial cells (38) is considered irrelevant, measurement of BDNF levels in peripheral blood in equivalent to BDNF levels in the CNS (39).

A study found decreased levels of BDNF in platelets of 24 BPD patients compared to 18 healthy control individuals. In addition, this study compared 18 male patients that did not use any antipsychotic drugs for one month or any antidepressant medication for three weeks with 18 control group individuals (11 male patients) (40). In contrast, another study found increased BDNF levels in 115 BPD outpatients compared to 52 healthy controls. Of the BPD outpatients, 108 were female and 55 used antidepressant drugs (41).

Those results strengthen the importance of the correlation between BDNF and BPD. In addition, until the writing of this paper, only 2 clinical articles were found that correlated the dosing of BDNF in patients with BPD.

### BPD and inflammatory cytokines

Another hypothesis that explains the pathophysiology of BPD is related to neuroinflammatory processes that are linked to the immune system itself. The immune system, which is divided into an innate and an acquired system, interprets changes in the environment to create appropriate responses, identifying substances and organisms that are harmless or could be dangerous (42). The innate system represents the first line of defense and provides a fast response and recruitment of defense cells using cytokines and chemokines, which are the substrate of inflammation. In contrast, the acquired immune system can take up to 5 days to produce adequate responses.

Among various proteins produced to provide immune protection, cytokines represent a broad group that may be produced by cells of the immune system - innate (granulocytes, monocytes, macrophages, dendritic cells, and natural killer cytotoxic cells) or acquired (helper T cells, cytotoxic T cells, and B cells) (43). Depending on the response, they can produce an inflammatory or anti-inflammatory response.

However, it was not until the discovery of techniques that enabled cytokine detection in the 20th century, such as flow cytometry (44), that it was possible to investigate psychiatric immunology and find an association between an imbalanced immune system, cytokines, and mental disorders (45). After this, research on inflammatory factors in mental disorders has noticeably grown over the last decade. One of those findings described the correlation between inflammation and microglial and astrocyte activation in schizophrenia, depression, anxiety, and autism spectrum disorders. Inflammatory processes in these diseases seem to be related to several pathways, including kynurenine (46).

In the CNS, the tryptophan-kynurenine pathway starts with the conversion of tryptophan into kynurenine by indoleamine 2,3-dioxygenase 1 (IDO1), IDO2, or tryptophan 2,3-dioxygenase (TDO). Then, kynurenine can follow two metabolic branches: one implicated in neuroprotection, producing NMDA and α7-nicotinic acetylcholine receptor antagonist or one related to neurotoxicity, with production of a NMDA receptor agonist that leads to oxidative stress (47,48). Inflammatory stimuli lead to upregulation of tryptophan-kynurenine pathway that generates neurotoxic and pro-apoptotic catabolites, probably worsening BPD symptoms.

The need for research on this area is evident, mainly because there are only two published studies involving cytokines in patients with BPD. The first study included 12 female patients with BPD, without any medication for 8 weeks, showing an increased level of inflammatory cytokines, such as TNF-α and IL-6 (49) compared to a healthy group of 12 female individuals. The second study included 38 female patients with BPD, 11 of which were using serotonin reuptake inhibitor (SRI) to treat nervous bulimia or depression, and found increased levels of TNF-α and IL-6 (50) compared to 22 healthy women. These findings demonstrate the importance of understanding the link between BPD and inflammation.

### BPD and oxidative stress

In the search for possible biomarkers for BPD pathophysiology, the interaction between BPD and oxidative stress was explored in this study. The topic has gained importance in the fields of biochemistry, medicine and life sciences (51).

The concept of oxidative stress was introduced to the scientific community over 30 years ago (52). Oxidative stress results from an imbalance between oxidant and antioxidant chemical species, leading to a dysfunction of redox signaling and cell physiology (53). Redox signaling acts through reversible modifications on cysteine residues, induced by reactive oxygen species (ROS), which target proteins within kinase cascades, ion channels, apoptotic cascades, and phosphatases (54,55).

However, ROS, which are by-products of aerobic metabolism that include substances such as superoxide anion (O_2_^-^), hydroxyl radicals (OH^-^), and hydrogen peroxide (H_2_O_2_), are no longer considered only substances that cause damage to lipids, carbohydrates, proteins, and DNA (56). Now, they are also considered signaling molecules (57) that are important in physiological and biochemical processes of the human body.

Thus, it is not the presence of ROS, but its excess that can cause irreversible and harmful chemical changes in the body (57). As an example of this negative role, lipid peroxidation, a reaction that results from the interaction of ROS and lipids, produces malondialdehyde (MDA), a biomarker of oxidative stress that is measured by reactions with thiobarbituric acid (TBARS) (58). In animal and human studies (59,60), TBARS have been used to evaluate levels of oxidative stress. In addition, other substances that may reflect oxidative stress level are glutathione (GSH) (61), catalase, and superoxide dismutase (62).

ROS are produced mainly by mitochondria (63) and may result from physiological processes or pathological conditions. Despite the enzymatic and non-enzymatic defense systems, ROS accumulation may occur and seriously affect some parts of the body, especially the CNS, which is very dependent on oxygen. The dysregulation of redox signaling in the CNS leads to neurodegeneration and may contribute to neuropsychiatric diseases (10).

Although it is already a consensus in the scientific literature that changes in MDA and glutathione (GSH) (64) are responsible for brain changes in some mental disorders (65), there are only 2 studies evaluating oxidative stress in BPD. One of them included 43 BPD patients (27 females, with a mean age of 36.3 years), and found an increase in 8-hydroxy-2-deoxyguanosine (8-OH-DG), the oxidized form of guanine, in peripheral blood, which indicated a rise in oxidative stress (66).

The other study included 49 BPD patients (43 females with mean age of 29.8 years) and aimed to analyze oxidative and inflammatory responses and their correlation with clinical findings. The results show a decrease in antioxidant enzymes (catalase, dismutase, glutathione peroxidase) and an increase in TBARS in BPD patients. In addition, there was a rise in NFκB, iNOS, COX2, Keap1, and NQO1 inflammatory factors and a decline in IκBα levels in those patients (67). The patients had a mean of 17 years of BPD diagnosis, 34% used antipsychotic and antiepileptic drugs, and more than 65% used antidepressants and benzodiazepines.

Therefore, different oxidative stress patterns are present in BPD. The importance of such patterns for pathophysiology still needs to be established by future research. A possible explanation is that oxidative stress may be induced by inflammatory response (62). As with BPD, a condition that leads to high inflammatory markers, it is understandable that oxidative stress can lead to an increase in inflammatory markers in other pathologies. On the other hand, inflammation can be triggered or enhanced by oxidative stress through activation of nuclear factor-kappa B (NF-κB), which controls the expression of many genes, including inducible nitric oxide synthase (iNOS) and cyclooxygenase 2 (COX2), which are involved in proinflammatory cytokine production (68). This bidirectional connection between inflammation and oxidative stress may be involved in BPD neurobiology (67).

Furthermore, prospective studies have shown that the use of antioxidant compounds in mental disorders seems to improve disease symptomatology (69,70). A recent study of the Neuropsychopharmacology Laboratory of the Federal University of Ceará (UFC) described that α-lipoic acid, a powerful antioxidant with anti-inflammatory effects, decreased lipid peroxidation levels and improved measurements of psychopathology when used in 10 patients with schizophrenia (71). This result suggests that oxidative stress and inflammatory markers play an important role in neural damage in BPD.

### Conclusions

BPD is a health problem that has a significant prevalence in the population. However, more research on its pathophysiology is still needed to seek effective pharmacological treatments and reduce the stigma and dysfunction faced by patients.

The results of this paper show that alterations in BDNF, inflammatory cytokines, and oxidative stress are still rarely studied in BPD, indicating that the search for specific biomarkers and molecular pathways for this mental disorder are still in the very beginning.

Understanding the relationship between BPD, inflammation, and oxidative stress may support future findings on the use of antioxidants or anti-inflammatory agents as adjuvants in the treatment of mental disorders.
